# Clinical Experiences in Private Practice, from a Physical Basis

**Published:** 1870-07

**Authors:** Z. C. McElroy

**Affiliations:** Zanesville, Ohio


Article II.—*Clinical Experiences in Private Practice; from a Physical Basis of Life.* By Z. C. McElroy, M.D., Zanesville, Ohio.

Albert Patterson, aged 12 years; comfortably housed, clothed and fed; goes to school during the day, and serves a carrier's route on a daily evening paper; returned from school, Wednesday afternoon, 30th March, 1870, and complained to his mother that his head ached, and that he felt tired. She noticed that his face was flushed, but attributed both to a cold. He, however, served his carrier's route that and each succeeding evening during the week. On Sabbath he was, if anything, worse, and seemed reluctant to go to sabbath school; but a few cheering words from his mother induced him to go. At sunday school he was very dizzy, and thought many times he would have to lie down, but managed to stay till school was dismissed. He reached home only with the greatest difficulty, was very dizzy, tired and weak, and went to bed immediately. His mother bathed his feet in hot mustard water, and as they were still cold, placed hot irons to them in bed. As he was no better on Monday, his mother packed him in a sheet, wet with warm water, during the forenoon, for the purpose, as she said, of breaking up his cold. He was in the wet sheet about an hour and a half, and on taking him out, his mother made an attempt to wash him all over, with hot water, in which she had dissolved some washing soda, but he became very sick, had a sinking spell, and she thought he would have convulsions. She rubbed him with warm flannel, and hastily put him to bed. He now became delirious all the time, and his hands and feet could not be kept warm.

Such is the mother's statement of his condition previous to my first visit, 6½ o'clock, P. M., Monday, 4th April, 1870. Skin cold, purplish, and clammy; pulseless at both wrists; just below the axilla, on the inside of the arm, the pulse counted 180; pupils dilated; temperature 107; shallow, rapid, and sighing breathing. Could get no response to questions, either by myself or his mother. He was in fact, and to all intents and purposes, in articulo mortis. In my first, and necessarily hurried investigation of Albert's case, no effort was made to connect the phenomena with the name of any disease, nor were any questions asked of the parents, in regard to probable causes. Fully recognizing that there are but three possible processes in any living body, viz: increased motion in the interest of repair, or waste; decreased motion in the interest of repair, or waste; and changing forms; no difficulty was encountered in getting at what was actually taking place in little Albert's body, without coupling the pathological condition with any name for his condition, which was that motion in the interest of repair was wholly suspended, while motion in the interest of decay, or waste, was proceeding at a velocity only co-incident with changes in the molecular forms, or structure of his tissues, with retention of the results of tissue metamorphosis. The indications for remedial management were to retard motion, eliminate effete matter, and change the chemical nature of the virus, or poison, so-called, or rather the mode of force engaged in changing the molecular forms of the tissues.

Impressed by the imperfect circulation of his blood, that there was a large deficiency of water in his body, it was decided to flood it with water, the effect of which would be a probable return of the circulation to the extremities, possibly some of it would be returned by vomiting, but the greater part would find exit through the kidneys, carrying with it some of the results of tissue metamorphosis, and would certainly open the way for more efficient elimination by all the avenues through which effete matter is passed from the body. In his unconscious condition this was no easy task, as all he would probably do would be to resist its introduction. A pitcher of warm water and tumbler were brought to me, and, aided by his mother and sister, and a resolute neighbor lady, some eight or ten tumblersfull were passed down his throat, and to the whole proceeding he made all the resistance in his power. To a looker-on the process of compelling the child to swallow that which, apparently, he did not want, would appear an unnecessary and cruel proceeding with a dying boy. But the stake was a large one—a life to be won or lost in a very few hours. At the conclusion, when he was laid down, the pulse had returned, as anticipated, in considerable volume, at his wrists. But his extremities were still cold, and requesting warm irons to be placed to his feet and legs, he was left for a couple of hours.

At 9½ P. M. found Albert very restless, pulse small and thready at wrists, and flitting at 180 per minute; he had not vomited any; his temperature was not ascertained. Half an ounce of Chlorate Potassa was dissolved in one pint of water, patient to have a wine-glassfull every two hours till gone. Requesting his mother to have him placed on the vessel and asked to pass water, at least every two hours, and to keep warmth to his feet, he was left for the night. Before leaving the house the mother and family were informed of his desperate condition, and that though he would probably die during the night, it was my desire that she should resolutely compel him to swallow the Chlorate solution, and not to omit anything she was requested to do, for it was barely possible that his youth and the treatment might carry him through the crisis.

On my way home, my mind, of course, dwelt on his case. Here was a little boy at the very verge of death, due, in the technical language of the profession, most likely, to some poison or virus. Scarlet fever, so-called, had been lingeringly prevailing in our community, for the past year, and the phenomena presented by little Albert impressed me with the idea that he was being overwhelmed by the so-called scarlet fever poison. The temperature indicated a rapidity of molecular changes incompatible with the maintenance of the normal molecular forms of the tissues for many hours; and as he had only ordinary evacuations from either bladder or bowels since the commencement of his illness, his body was loaded down with the results of tissue decay; and his immediate danger was, therefore, from so-called uraemic poisoning, which, in language which will give rise to correct mental impressions, is not simply urea, or urine retained in the blood, but the total results of tissue metamorphosis, less the gaseous products escaping at his lungs.

All organic poisons, so-called, produce their effects in living bodies by changing the molecular forms of the tissues; and any organic compound, solid, or fluid, or gaseous, which does not modify, or change tissue forms, is as innocent as milk, or water itself. And all organic poisons, so-called, but more properly form-changers, originate from the retrograde metamorphosis of animal tissues, or other complex organic compounds. Poison, organic poison, originates in decay, and is symbolical of decay. Living beings, more particularly those eating flesh, or concentrated food, have no greater enemies in nature than the results of the decay of their own tissues. The herbivora less so, but still more or less dangerous. The flesh-eating lower animals, for the most part, cover their excrement with earth.

But in reference to little Albert, with something over two quarts of water recently introduced into his body, with what he will get during the night, holding the Chlorate of Potassa in solution, elimination can hardly fail to be active during the latter part of the night, it seems to me he has had given to him the best chance for his life known to the practitioner's art.

5½ A. M. Patient much the same as at last visit last night; perhaps more restless, as he does not lie still five seconds at a time, the jactitation is extreme. Has consumed the whole pint of Chlorate Potassa solution, and his mother, fearing that he had lost some, had made another solution, one-half of which had been given. Had passed urine several times during the night, but has no pulse at the wrists; skin cold, clammy, and purplish; pupils widely dilated; temperature 106. Determined, if possible, to retard the velocity of tissue waste, and to bring on a critical elimination, gave ten drops Fluid Extract Veratrum Viride, to be repeated every hour till my return. Determined, also, to give him every possible chance for his life, the injection of liquor ammonia into one of his veins, was the next step resolved on. A neighboring physician was called on to assist, but not finding him at home, the matter was unavoidably postponed till after breakfast.

8 o'clock, A. M., 5th April. Albert has had three doses Veratrum; the pulse had fallen to 84 per minute, and could now be felt at the wrists; temperature 103; very restless; bowels discharging involuntarily in bed. In consultation, the case was considered hopeless, and as the pulse had fallen, the injection of liquor ammonia was deferred, for the reasons that to get at a vein, a wound must be made, and as the proceeding was unusual, in case of his death, there might be censure. The consulting physician stumbled me no little by stating that he thought his lungs were so much congested as not to permit the air to enter. Hastily returning to the patient, they were examined, but found entirely free, and the air entering all parts of them. The consulting physician also objected to Veratrum, on account, as he said, of its depressing effects. I suggested Chloral Hydrat, to which assent was given, and a two ounce solution, with drachm of Chloral, prescribed, and sent for; and a tablespoonfull to be given every hour till restlessness was controlled. A flax-seed cataplasm, with one-eighth ground mustard, was prescribed, to cover his chest, and a mustard poultice, alone, to nape of neck. He is also to have brandy and milk forced down his throat. A previous engagement for this day would prevent my seeing Albert again till evening, the consulting physician was to call at noon. He found he had vomited very freely, and had passed a good deal of urine and feces in bed, and was much more calm. The Veratrum was resumed in small doses every hour.

5 o'clock, P. M. Temperature 103½; pulse 120; much better. He sleeps some now, and begins to demand fluids. He is to have solution Chlorate Potassa during the night, and bread and milk, if he will take it.

6th April, 8 o'clock, A. M. Has slept some; is quite rational; passes urine freely; temperature 102½; pulse 120. Is considered convalescent; to have food, and, in the afternoon, Rochelle Salts, to move his bowels.

A report reaches the family that one of the little carrier boys, while waiting for their papers, Saturday afternoon, at the printing office, had some wintergreen leaves, or, as he called it, mountain tea, which he gave to the other boys; that Albert had taken some, and on tasting them said they were very bitter, and it was soon ascertained that they were Laurel leaves. (Kalmia Latifolia). But Albert, who is now entirely rational, says he did not taste any, nor had he any at any time in his possession. He remembers that there was a boy who had some mountain tea, but he did not give him any; and persists in saying so to the present time. The symptoms of Laurel poisoning would hardly be delayed so long, and besides, they indicate that Laurel only retards motion, but does not change forms, so that his statement that he did not eat any is probably correct. He does not now remember anything after returning from sunday school.

7th April, 9 o'clock, A. M. Has slept a good deal, and is still improving. Says he feels weak, but has no pain, and takes considerable food. Pulse 120; temperature 102½. Bowels not having moved, he is to have solution of Epsom Salts, this forenoon, and all the food he will take.

5 P. M. Bowels have moved freely; temperature 103½; pulse 120. To have Chlorate Potassa solution during the night.

8th April, 9 A. M. Very much better again this morning; enjoys his food; passes water freely, but has a temperature of 102½, with pulse 116. As motion in the interest of repair is fully active, nothing to be done to retard the rate of waste in progress, as shown by the thermometer.

9th, A. M. Still improving, eats well, sleeps well; wants to be dressed and set up to-day, and is permitted to do so. Temperature 102; pulse 116.

The further progress of the case presented only this special feature, that for ten or twelve days next succeeding, his temperature and pulse, whenever ascertained, and that was only once a day, or on alternate days, was never below 102, and 114. Had the process of repair been less active, this would have occasioned some anxiety; but as motion was equally active in the interests of both repair and waste, no interference was made by remedial agents. In ten or twelve days he resumed his duties on the carrier's route, and calling at my office occasionally, his temperature and pulse were never found below 102 and 110.

It still seems to me, Albert was poisoned, in professional technology; that the greater part of his body had undergone such changes of molecular forms, by some mode of force, probably that of so-called scarlet fever, as to render it unfit for the evolution of the normal phenomena of life. But it matters very little in the professional management of his case, what particular mode of force it was; as the result and management would have been the same. The pathological fact was, that when first seen, motion in the interest of repair was wholly suspended; and the rate of waste was rapidly hastening him down to death. In looking backwards at the case, it would appear as probable that flooding his system with warm water, the Chlorate Potassa, and Veratrum Viride, were the agents which turned the tide of his life. The Chloral Hydrat was certainly inert, and he owes his life to the thorough elimination, mainly brought about by the water and Chlorate Potassa, and the sudden check of motion in the interest of waste, by the Veratrum. The five or six drachms of Chlorate of Potassa given the first night may seem large, and it was large, but none too large, for it seems to me the results justify the means to the end attained. Each of these agents was employed with definite conceptions of the processes and ends to be accomplished; and from beginning to end, there was no doubt or hesitation, save the little jolt due to the consultation.

The management of this case may be taken as an example of therapeutics from a physical basis of life, and can hardly fail to commend itself to practitioners most indifferent to "new theories," as they term them, in reference to pathology and therapeutics as well as physiology.*

* See Chicago Medical Journal, January, 1870.

